# Influence of the static magnetic field on cell response in a miniaturized optically accessible bioreactor for 3D cell culture

**DOI:** 10.1007/s10544-019-0387-8

**Published:** 2019-03-13

**Authors:** Luca Izzo, Marta Tunesi, Lucia Boeri, Matteo Laganà, Carmen Giordano, Manuela Teresa Raimondi

**Affiliations:** 1 Department of Chemistry, Materials and Chemical Engineering “G. Natta”, Politecnico di Milano, Piazza Leonardo da Vinci 32, 20133 Milan, Italy; 2 Gemma Prototipi Studio, Erba, Italy

**Keywords:** MOAB, Static magnetic field, Perfusion bioreactors, Numerical characterization, 3D cell culture, Cell metabolic activity, Gene expression

## Abstract

Hydraulic sealing is a crucial condition for the maintenance of sterility during long term operation of microfluidic bioreactors. We developed a miniaturized optically accessible bioreactor (MOAB) allowing perfused culture of 3D cellularised constructs. In the MOAB, the culture chambers are sealed by magnets that generate a weak static magnetic field (SMF). Here, we predicted computationally the exact level of SMF to which cells are subjected during culture in the MOAB and we assessed its influence on the viability, metabolic activity and gene expression of neuroblastoma-derived cells cultured up to seven days. The predicted SMF ranged from 0.32 to 0.57 T using an axial-symmetric model of a single chamber, whereas it ranged from 0.35 to 0.62 T using a 3D model of the complete device. Cell function was evaluated in SH-SY5Y neuroblastoma cells at 2 and 7 days of culture in the MOAB, compared to 2D monolayer, 3D non-perfused constructs, and 3D perfused constructs cultured in a modified MOAB with magnet-free sealing. We measured the cell metabolic activity normalized by the DNA content and the expression levels of heat-shock protein 70 (Hsp-70), Bcl-2 and Bax. We found that the level of SMF applied to cells in the MOAB did not influence their metabolic activity and exerted a stressful effect in 2D monolayer, not confirmed in 3D conditions, neither static not perfused. Instead, the magnets provided a significantly greater hydraulic sealing in long-term culture, thus the MOAB might be potentially exploitable for the development of reliable *in vitro* models of neurodegeneration.

**Electronic supplementary material:**

The online version of this article (10.1007/s10544-019-0387-8) contains supplementary material, which is available to authorized users.

## Introduction

*In vitro* cell culture on two-dimensional (2D) glass or plastic substrates fails to model the complex physiological three-dimensional (3D) environment, enable cell differentiation and reproduce *in vivo* cell behavior; furthermore, the absence of a continuous flow of medium does not provide a continuous nutrient supply and waste removal (Lv et al. 2017). Oppositely, *in vitro* cell modelling with miniaturized bioreactors shows great advantages. It requires small volumes of reagents and cells, both critical elements for valuable samples and high-throughput screening. Portability, design versatility, potential for parallel operations and integration with existing devices or platforms are further advantages (Lübberstedt et al. 2015). For instance, Lei and colleagues (Lei et al. 2014) provided an interesting example of a microfluidic chip coupling both 3D microenvironment and perfusion by perfusing human oral cancer cells embedded in an agarose gel at a flow rate of 10 μL/h in a culture chamber of 4x2x1 mm.

Optical accessibility represents a considerable improvement, since it allows for the reduction of samples and the analysis of the same constructs with time by non-destructive techniques like viable staining and standard fluorescence microscopy. As an example, Kim and co-workers (Kim et al. 2012) applied a miniaturized optically accessible perfusion bioreactor with the possibility to host 3D tissues for *in vitro* modelling of the human gut.

**Fig. 1:**
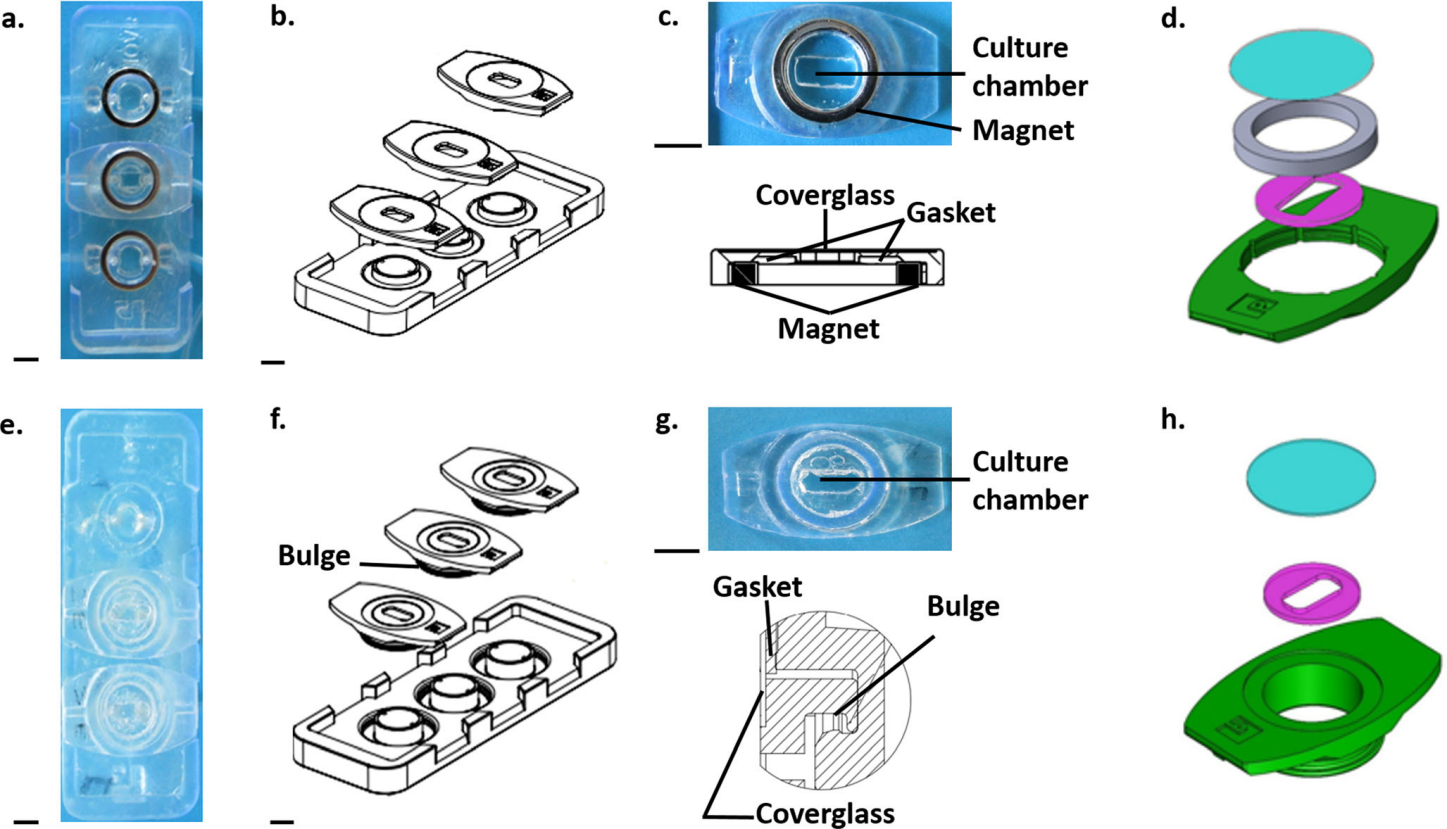
**Views and technical sketches (AutoCAD® Software, Autodesk, San Rafael, California, USA) of the miniaturized optically accessible bioreactor (MOAB) with magnetic (upper panel) or snap-fit closure (lower panel). a** Top view of the MOAB with magnetic closure. **b** Technical sketch showing the MOAB with magnetic closure. **c** Top: top view of one culture chamber with magnetic closure; Bottom: side sketch of one culture chamber with magnetic closure. **d** Technical sketch showing the lid (green), the gasket (purple), the magnet (grey) and the glass coverslip (light blue). **e** Top view of the MOAB without magnetic closure. **f** Technical sketch showing the MOAB without magnetic closure. **g** Top: top view of one culture chamber without magnetic closure; Bottom: side sketch of one culture chamber without magnetic closure. **h** Technical sketch showing the lid (green), the gasket (purple) and the glass coverslip (light blue) in the MOAB without magnetic closure. Scale bar 5 mm

In this context, Laganà and Raimondi (2012) developed a miniaturized optically accessible bioreactor (MOAB) for the interstitial perfusion of 3D cell constructs. To simplify the assembly procedure while reducing the time required, we have optimized their initial prototype. The current device (Fig. 1, top) is composed of three independent and magnetically lockable chambers (9 mm^3^) assembled on the top surface of a common main body (68 × 25 mm) made up of medical grade polystyrene. The main body has rigid edges to reduce the optical path and enhance sample illumination during optical transmission microscopy. The first magnet (NdFeB ring magnet, 12 mm outer diameter, 9 mm internal diameter, 1.5 mm thick) is located in the chamber, while the second one is in the bioreactor body. Their magnetic coupling (closure force of 14.7 N) ensures the hydraulic sealing during the perfusion of 3D constructs (6x3x0.4mm) and simplifies the assembly procedure, allowing for the self-centering and self-aligning of the locking system with respect to the feed channels. Each chamber has a 9 mm glass coverslip equipped with a medical grade silicone gasket. It prevents the movements of the constructs by mechanical interference. By changing the shape of the gasket, it is possible to host differently shaped constructs (e.g. scaffolds, hydrogels) in the culture chambers. The whole device may be sterilized with hydrogen peroxide gas plasma systems (e.g. STERRAD® 100S, ASP, Jonhson & Jonhson, Irvine, CA, USA). The MOAB is a versatile and tunable platform, already validated for advanced *in vitro* cell modelling in several research fields, including neuroscience (Tunesi et al. 2016) and cancer (Marturano-Kruik et al. 2018). Thanks to its optical accessibility, it was also applied for *in vitro* tracking of exosomes in a model of gene therapy for muscular dystrophy (Frattini et al. 2017).

The magnets sealing the culture chambers of the MOAB generate a static magnetic field (SMF) that might influence cell functions. SMFs interact with biological systems by both electrodynamic and magneto-mechanical effects, meaning that moving ionic charges induce an electric potential because of the Lorentz force and both diamagnetic and magnetic substances are exposed to a torque and orient, respectively (Roth 2011). SMFs are naturally present: the Earth itself generates a magnetic field ranging between 25 and 65 μT and involved in the orientation and migration of some animal species (Feychting 2005). Industrial activities exploiting direct currents also generate SMFs (e.g. in aluminum production or chloralkali plants, workers are exposed to SMFs varying from 4 to 50 mT (European Commission 1996)). Therapeutic devices (e.g. prosthetic cardiac pacemaker, defibrillator, standard orthodontic devices, Holter) also produce SMFs up to 10 mT. In particular, defibrillators and standard orthodontic devices expose the patients to a SMF for very short periods, but the operators for frequent times. For medical and research purposes, stronger SMFs may be applied. For instance, for magnetic resonance imaging (MRI) the magnetic field can reach intensities up to 3 T (Hartwig et al. 2018), while the operators are exposed to a magnetic field varying from 0.5 to 2 mT (Zannella 1997).

Valiron and colleagues (Valiron et al. 2005) observed that exposure to high magnetic fields (over 10 T and 15 T for cycling cells and neurons, respectively) for 30–60 min affects cell cytoskeleton, with deleterious effects on cell viability (e.g. detachment from culture dishes), organization and differentiation. A reduction in cell viability was also reported when decreasing the intensity of the SMF and increasing the exposure time. For example, Raylman and co-workers (Raylman et al. 1996) obtained a reduction in the number of viable HTB 63, HTB 77 IP3 and CCL 86 malignant human cell lines placed for 64 h in the isocenter (~5 cm diameter) of a superconducting solenoid magnet generating a 7 T uniform SMF. Similarly, Ji and colleagues (Ji et al. 2009) observed a decrease in the number of *E. coli* colony-forming units after exposures up to 60 min to two different permanent magnets (magnetic field induction between 45 and 450 mT and 0.45–3.5 T. In the latter configuration, the maximum space for exposure was 17x17x10 cm). To study the effects of the intensity of the SMF that patients are exposed to during MRI, Zhang and co-workers (Zhang et al. 2017) plated 15 different cell lines on the top surface of a magnet (1 T, 5x5x5 cm), reporting that the SMF affects cell proliferation in a cell type- and density-dependent manner. The dependence on cell type was also suggested by Aldinucci and colleagues (Aldinucci et al. 2003), showing that the combination of a static electromagnetic field (4.75 T) with a pulsed electromagnetic field (0.7 mT) generated by an NMR apparatus had neither proliferative, nor activating or proinflammatory effect on lymphocytes after 1 h-exposure, while it affected proliferation in Jurkat cells.

This work takes place inside two novel technological projects, named MOAB (ID number 825159) and MINERVA (ID number 724734) supported by the European Research Council (ERC) Programme. In particular, MINERVA project aims, by using an innovative bioengineering approach, at evaluating the potential impact of human gut microbial community on central nervous system functionality also in neurodegenerative disorders. MINERVA goal is to develop a cutting-edge technological platform, based on organ on chip microfluidic device, to model the main players of the microbiota-brain axis connection. In this context, with a view to exploit the improved version of the MOAB also as the basic functional unit for the development of reliable *in vitro* models of neurodegeneration in MINERVA project, in this work we focused on predicting the SMF generated by its magnets and measuring its effects on viability, metabolic activity and gene expression in SH-SY5Y neuroblastoma cells, a commonly used cell model in the research against neurodegeneration. As a control, we used MOABs with a non-magnetic lock (e.g. snap-fit closure, Fig. 1, bottom), a custom-made version of the device where the magnets are not present and the hydraulic sealing is assured by the mechanical interference between the lids (showing a bulge) and the main body of the bioreactor.

## Materials and methods

### Numerical prediction of the level of magnetic field

To study the distribution of the intensity of the SMF generated by the magnetic closures, we performed a numerical analysis with the software COMSOL Multiphysics® (Burlington, MA, USA), version 5.2. We exploited both an axial-symmetric and a 3D complete model and compared their results. In the first model, we considered only one chamber (thus hypothesizing that they are independent), while in the second one we included all the three chambers (thus taking into account their possible reciprocal influences).

**Fig. 2:**
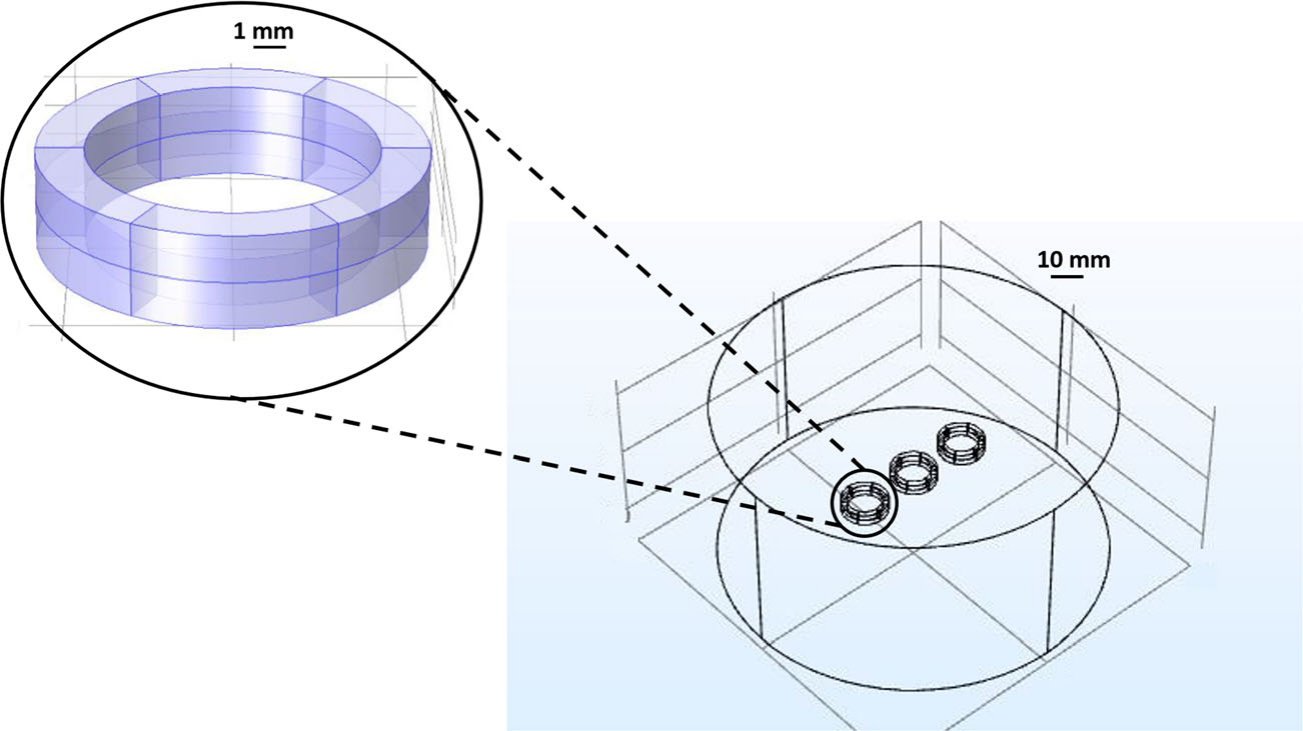
**COMSOL Multiphysics® modeling of the magnetic coupling of the MOAB.** Geometry of the COMSOL Multiphysics® models for the three chambers of the MOAB. Left: Enlargement of a single couple of magnets and geometry of the exposure zone. Right: To visualize the distribution of the intensity of the static magnetic field, we assumed an air-made cylinder with two coupled neodymium-based ring magnets in the isocenter (axial-symmetric model) or six coupled neodymium-based ring magnets (two in the isocenter and four 16 mm far, 3D complete model)

The geometry of the 3D complete model (Fig. 2) was represented by three couples of permanent ring magnets (Supermagnete, Gottmadingen, Germany) identical to those in the MOAB. We reported their properties in Table 1. We chose neodymium as the material for the domains of magnetic coupling. To simulate the external environment and visualize the distribution of the magnetic field intensity around the chambers, we assumed an air-made cylinder of 50 mm in diameter and 50 mm in height, with a magnetic insulation for the external surface. Its isocenter corresponded to the center of the central magnetic chamber; while the centers of the two lateral chambers were 16 mm far. This distance was in accordance with the geometry of the MOAB. We chose the dimensions of the cylinder to observe a null value of magnetic field intensity on its boundaries.

**Table 1:** Properties of NdFeB magnets

Property	Value
Material	NdFeB
Cross section shape	Rectangular
Outer diameter	12 mm
Internal diameter	9 mm
Height	1.5 mm
Tolerance	± 0.1 mm
Closure force	14.7 N
Direction of magnetization	AXIAL (that is parallel to the height)
Coating	Ni-Cu-Ni
Magnetization	N45
Weight	0.56 g
Residual magnetism	1.37 T
Max temperature	80 °C

List of the features of each ring magnet (Supermagnete, Gottmadingen, Germany) integrated in the microfluidic optically accessible bioreactor (MOAB) with magnetic closure

The properties of neodymium and air were already present in COMSOL Multiphysics® database, except for the relative permeability.

For both models, we inserted a value of 1.04 and 1 for neodymium and air, respectively. In addition, the Maxwell equations modelling the physical phenomenon were already present in the tool *Magnetic Fields No Currents*:

1.1 $$ \mathrm{H}=-\nabla {\mathrm{V}}_{\mathrm{m}} $$

where H is the magnetic field intensity and V_m_ is the magnetic scalar potential;

1.2 $$ \nabla \cdotp \left({\upmu}_0{\upmu}_{\mathrm{r}}\mathrm{H}+{\mathrm{B}}_{\mathrm{r}}\right)=0 $$

where μ_0_ is the vacuum permeability, μ_r_ is the relative permeability of the material and B_r_ is the residual magnetism (assumed 2.74 T, that is 1.37 T multiplied by two, since we considered two coupled magnets). Other input parameters assumed were the temperature (293.15 K) and the absolute pressure (1 atm). An axially magnetized magnetic couple was present in the problem; therefore, we selected the correct domains to align the magnetized faces perpendicularly to the axial direction. We also assumed absence of radial magnetization. To estimate the intensity of magnetic induction field as a function of the distance from the symmetry axis of the magnet(s), for both models we chose the option *very dense mesh* for both the magnet(s) and the air-made cylinder. We extracted the results of both models in a colour map showing the distribution of the magnetic induction field around the magnet(s).

### Hydraulic characterization of magnetically and non-magnetically lockable MOABs

#### Static leakage tests

To assess the capability of both magnetically and snap-fit-lockable MOABs to face a pressure increase, we performed static leakage tests. We used a pressure regulator to provide pressure steps of 0.02 MPa to a distilled water reservoir connected to the inlet channel of each culture chamber by gas permeable tubes (internal diameter: 0.03″, outer diameter: 0.065″; Silastic® Tubing, Cole-Parmer, Vernon Hills, IL, USA). We tested the three chambers one by one, while clamping the outputs to allow for the pressure increase to the critical value. We maintained each pressure step for 30 s. We measured the maximum pressure value when the first water drop started coming out from the examined chamber. We tested two magnetically lockable and two snap-fit lockable MOABs and repeated the test three times for each chamber.

#### Hydraulic resistance

**Fig. 3:**
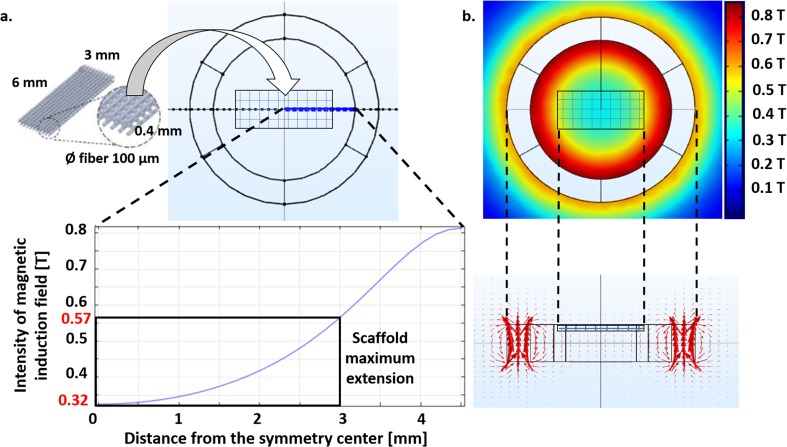
**Axial-symmetric model of the magnetic field generated in a single culture chamber. a** Intensity of magnetic induction field with the distance from the symmetry center of the magnets (blue line). The black rectangle highlights the dimensions of polystyrene scaffolds (6x3x0.4 mm) identical to those for cell experiments. They are obtained by fused deposition modeling and composed of four layers of fibers (diameter: 100 μm, with a pore size: 300 μm) shifted of 150 μm with respect to the adjacent. A sketch of the positioning of the scaffolds in the culture chambers is also shown. **b** Color map showing the distribution of the magnetic induction field around the magnets (top view) and side view of the arrow field. A sketch of the positioning of the scaffolds in the culture chambers is reported

To characterize the hydraulic resistance of the fluid pathway, we used a precision pressure regulator to provide pressures from 0.02 MPa to 0.1 MPa (with steps of 0.02 MPa) to a distilled water reservoir connected to the inlet channels of the culture chambers. We connected the output channels to a reservoir placed on a precision electronic balance (AP250D, Ohaus, Greifensee, Switzerland) and tested the three chambers one by one. We maintained each pressure step for 30 s, then we clamped the inlet channel and calculated the flow rate from the measured weight and the perfusion time. We tested two magnetically lockable and two snap-fit lockable MOABs and repeated the test three times for each input pressure value. To account for their hydraulic resistance, each chamber hosted a polystyrene scaffold (6x3x0.4 mm, 3D Biotek, Hillsborough, NJ, USA; Fig. 3, top) identical to those for cell experiments. It showed four layers of fibers (diameter: 100 μm, pore size: 300 μm) shifted of 150 μm with respect to the adjacent one.

#### Cell culture

We cultured SH-SY5Y human neuroblastoma cells (Izsler, code BS TLC 232) at 37 °C and 5% CO_2_ in high-glucose Dulbecco’s modified Eagle’s medium supplemented with 10% (*v*/v) fetal bovine serum, 2 mM L-glutamine, 100 U/mL penicillin, 0.1 mg/mL streptomycin sulfate (ThermoFisher Scientific, Waltham, MA, USA) and split twice a week. We examined the effects of the magnetic field generated by the NdFeB ring magnets in both 2D and 3D (polystyrene scaffolds) conditions.

#### Influence of the magnetic field on cells in 2D culture

We studied the influence of the SMF on SH-SY5Y cells in 2D culture after both short (48 h) and longer exposures (7 days). For the first condition, we plated 93,750 cells/cm^2^ in the center of 35 mm glass bottom dishes with 7 mm diameter center openings (MatTek Corporation, Ashland, MA, USA); while for the others, we plated 31,250 cells/cm^2^. These concentrations allowed for having enough cells after 48 h and avoiding over confluence after 7 days.

Since our predictions suggested that the SMF generated by a single couple of NdFeB magnets is a reliable approximation of the SMF to which the cells are exposed in the culture chambers (regardless of their position in the MOAB), we performed experiments in 2D conditions by reproducing a single chamber in a custom-made setup, also suitable for confocal microscopy. To prevent the corrosion of the magnets when in contact with medium and in humidified atmosphere, we coated the magnets with High Temp Resin (Formlabs, Somerville, MA, USA). We disinfected the samples by 70% (*v*/v) ethanol, washed extensively with distilled water and sterilized by UV irradiation (254 nm) for 1 h. One day after seeding, we placed our setup in the center of the cell-seeded glass bottom dishes. As a control, we cultured SH-SY5Y cells in the absence of NdFeB magnets in the same glass bottom dishes.

After 48 h and 7 days, we evaluated cell metabolic activity by resazurin (Sigma-Aldrich, St. Louis, MO, USA) assay by measuring the fluorescence at 590 nm (excitation wavelength 560 nm, Infinite M200PRO, Tecan, Männedorf, Switzerland). At the end of this analysis, we assessed the number of viable SH-SY5Y cells in the samples. We detached the cells from culture plates by trypsin, diluted with Trypan blue dye and counted. For each condition, we counted at least four squares. Starting from a previous study reporting that every human diploid cell contains about 6.4 pg DNA (Dolezel et al. 2003), we exploited these results to calculate the total content of DNA and the specific metabolic activity (that is cell metabolic activity normalized to the DNA content of each sample).

On day 7, we performed the live/dead assay (ThermoFisher Scientific). Medium was removed, and cells were washed with phosphate-buffered saline before incubation with 0.5 μM ethidium homodimer-1 and 0.4 μM calcein AM. Finally, samples were imaged by a confocal microscope (Fluoview FV10i, Olympus, Tokyo, Japan).

On day 7, we also evaluated the effect of the SMF on these target genes: Heat Shock Protein 70 (Hsp-70), Bcl-2 and Bax. Hsp-70 is an anti-apoptotic molecule that regulates several steps of the apoptotic cascade (Kong et al. 2016), while Bcl-2 has a role in inhibiting cell death. Oppositely, Bax is a pro-apoptotic molecule (Pawlowski and Kraft 2000). We performed the analyses on samples previously subjected to resazurin assay. We extracted the total cellular RNA with miRNeasy Mini Kit (Qiazol™, Qiagen, Hilden, Germany), according to the manufacturer instructions. We evaluated the quality of the extracted RNA via spectrophotometry (ND-1000; NanoDrop™, ThermoFisher Scientific). We synthesized the cDNA starting from 500 ng of total RNA with the High-Capacity cDNA Reverse Transcription Kit (Applied Biosystems™, ThermoFisher Scientific). We used the cDNA to measure the relative mRNA expression levels of Hsp-70, Bcl-2 and Bax via quantitative real-time polymerase chain reaction (qRT-PCR) with TaqMan™ Reagents (Applied Biosystems™). We used 18 s rRNA as a reference gene. For each sample, we normalized the expression of target genes to the expression level of 18 s rRNA. Among the groups, we normalized the expression ratio of magnetic samples to the corresponding control.

#### Influence of the magnetic field on cells in 3D culture

We plated SH-SY5Y cells in polystyrene scaffolds, as reported (Tunesi et al. 2016). After seeding, we kept the scaffolds in low-attachment culture plates for 2 days, and then we evaluated the effects of the SMF on SH-SY5Y cells both in dynamic and static conditions. We examined the following situations:

1. Presence of the SMF, dynamic culture: we cultured the scaffolds for 48 h or 7 days in dynamic conditions (MOAB with magnetic closing, flow rate: 0.5 μL/min; PHD ULTRA programmable syringe pump, Harvard Apparatus, Holliston, MA, USA);
2. Absence of the SMF, dynamic culture: we cultured the scaffolds for 48 h or 7 days in dynamic conditions in the absence of the magnetic field (bioreactor without magnetic closing, flow rate: 0.5 μL/min);
3. Presence of the SMF, static culture: we cultured the scaffolds for 48 h or 7 days in the presence of the magnetic field in static conditions (inside the custom-made setup described in the previous paragraph);
4. Absence of the SMF, static culture: we cultured the scaffolds for 48 h or 7 days in the absence of the magnetic field in static conditions (inside a low-attachment culture plate).

After 48 h and 7 days, we repeated the analyses described for 2D cultures. We analyzed the mRNA expression levels of Hsp-70, Bcl-2 and Bax for groups 1, 3 and 4.

#### Statistical analysis

We reported the results as mean ± standard deviation (SD). We performed the statistical analysis with GraphPad Prism® software (GraphPad Software, La Jolla, CA, USA). We used two-way analysis of variance (ANOVA) followed by Tukey’s multiple comparisons test for comparisons among the groups and time frames, while we used one-way ANOVA followed by Dunnett’s multiple comparisons test for comparisons among the groups. The significance level was set at *p* < 0.05.

## Results

### Numerical prediction of the level of magnetic field

**Fig. 4:**
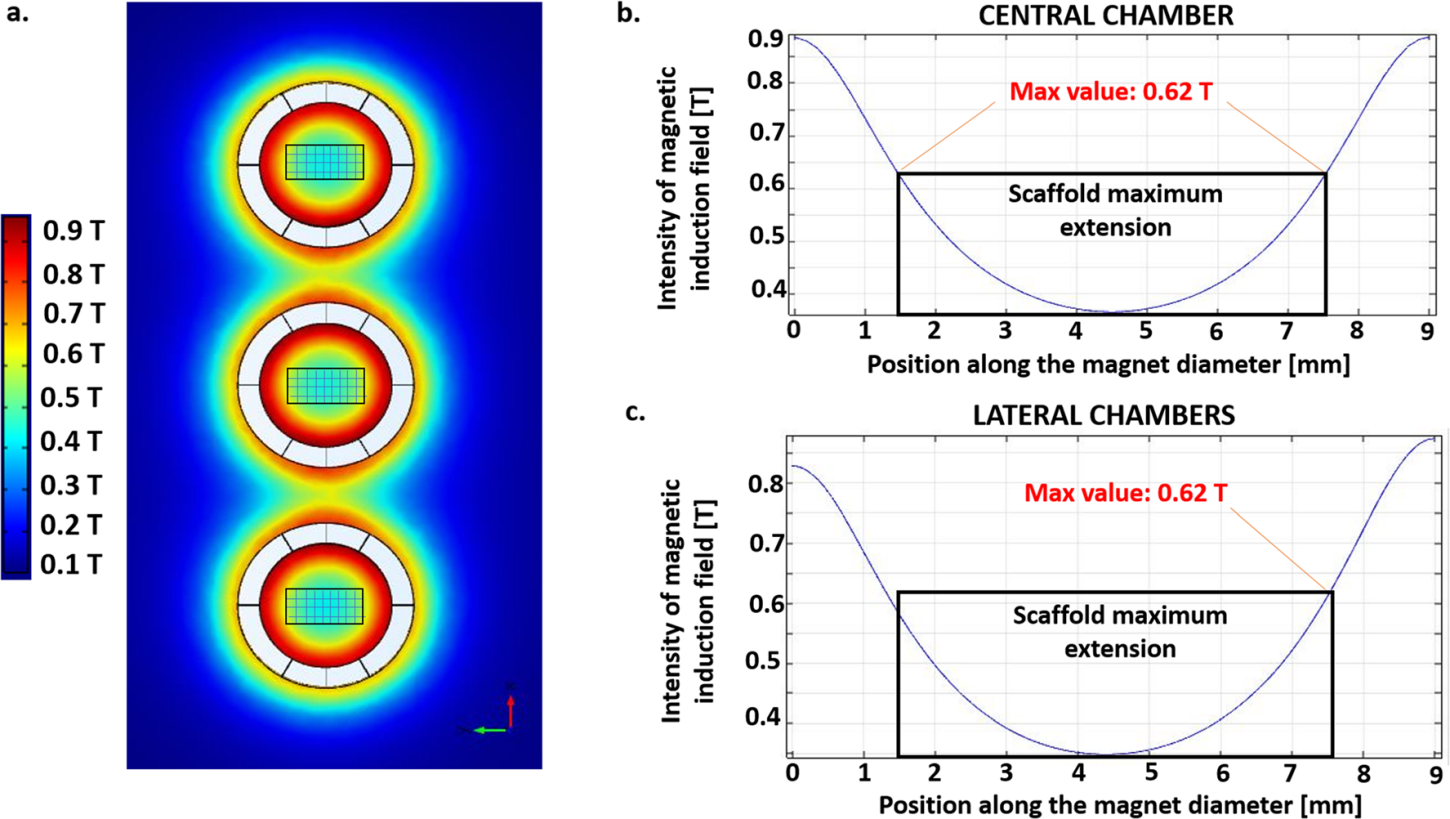
**Complete 3D model of the magnetic field generated by the three chambers. a** Color map showing the distribution of the magnetic induction field around the magnets (top view). The black rectangles highlights the dimensions of polystyrene scaffolds. **b** Intensity of magnetic induction field as a function of the distance from the symmetry center of the magnets for the central chamber (blue line). The maximum estimated value was 620 mT. **c** Intensity of magnetic induction field as a function of the distance from the symmetry center of the magnets for the lateral chambers (blue line). The maximum estimated value was 620 mT

Figure 3 and Fig. 4 show the results from the analysis with COMSOL Multiphysics® for the axial-symmetric and the 3D complete model, respectively. The black rectangle highlights the dimensions of the polystyrene scaffold within the culture chambers. For both models, according to Biot-Savart law, we observed a quadratic decay for the intensity of magnetic induction field in the area occupied by the scaffold. A magnetic field is present outside the rings, but its values rapidly reach zero while increasing the distance from the symmetry center.

With the axial-symmetric model, considering only one of the three chambers, we predicted that cell constructs are exposed to a SMF ranging from 320 mT to 570 mT. The arrow field lines showed the two magnetic poles of the coupling and confirmed the non-zero value of the magnetic field in the center of the culture chamber.

With the 3D complete model, considering all the chambers and their possible reciprocal influences, we predicted that the magnetic field intensity ranges from 350 mT to 620 mT in the lateral chambers and from 370 mT to 620 mT in the central one. Therefore, when the two other chambers are included in the model, in each lateral chamber the intensity of the SMF increases by less than 10%. For the central chamber, the minimum intensity of the SMF increases with respect to the lateral ones, but the maximum value is unchanged, suggesting that the influence of adjacent chambers is negligible. Therefore, a single couple of NdFeB magnets and an axial-symmetric model is suitable to estimate the intensity of the SMF in the scaffolds in the magnetically lockable MOABs with a reliable approximation.

Since our predictions have suggested that in the culture chambers cells are exposed to non-zero values of the SMF, effects on their viability, metabolic activity and gene expression might be observed.

### Hydraulic characterization of magnetically and non-magnetically lockable MOABs

#### Static leakage tests

**Fig. 5:**
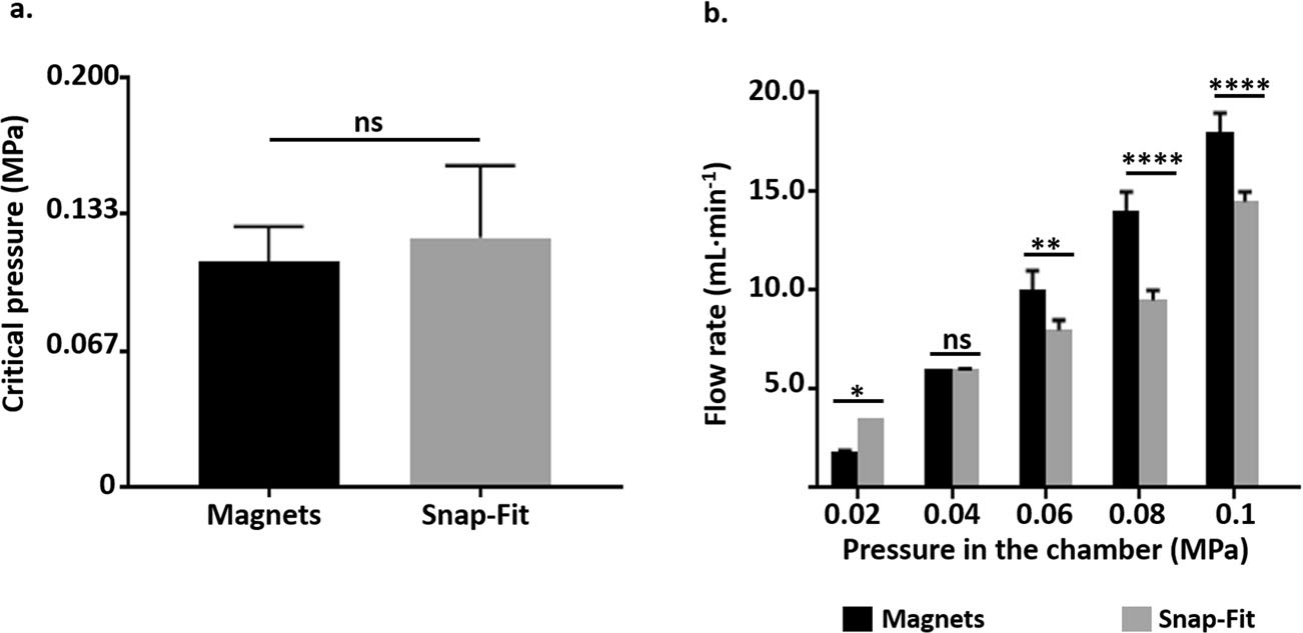
**Hydraulic characterization of MOABs with magnetic and snap-fit closing. a** Static leakage tests. We reported the results as mean ± SD (6 replicates/group). We performed the statistical analysis with t-test. ns, *p* > 0.05. **b** Hydraulic resistance. We reported the results as mean ± SD (6 replicates/group). We performed the statistical analysis with one-way ANOVA followed by Tukey’s multiple comparisons test. ns, *p* > 0.05; *, *p* < 0.05; **, *p* < 0.01, ****, *p* < 0.0001

Figure 5a shows the results from static leakage tests. We did not find any difference (ns, *p* > 0.05) between MOABs with magnetic and snap-fit closing, with values (mean ± SD) of (0.110 ± 0.017) MPa and (0.122 ± 0.035) MPa, respectively. In addition, for both MOABs we did not observe breaking while increasing the pressure.

#### Hydraulic resistance

Figure 5b shows the results related to the hydraulic resistance of the fluid pathway. For both magnetically and snap-fit lockable MOABs, the flow rate increased while raising the pressure. For a pressure of 0.02 MPa we observed a greater flow rate (mean ± SD) for the snap-fit bioreactors (1.8 ± 0.08 vs 3.5 ± 0.01 mL/min, *, *p* < 0.05); while for a pressure of 0.04 MPa we did not find differences between the two configurations (ns, *p* > 0.05). At this pressure, we recorded a flow rate of 6.0 ± 0.01 and 6.0 ± 0.04 mL/min for the magnetic and snap-fit MOAB, respectively. When we further increased the pressure, the magnetically lockable bioreactor offered a greater flow rate (**, *p* < 0.01 when we set pressure at 0.06 MPa; ****, *p* < 0.0001 when we set pressure at 0.08 and 0.1 MPa). At these pressures, we recorded a flow rate of 10.0 ± 0.82 and 8.0 ± 0.41 mL/min; 14.0 ± 0.82 and 9.5 ± 0.41 mL/min; 18.0 ± 0.82 and 14.5 ± 0.41 mL/min for the magnetic and snap-fit MOAB, respectively. These values are greater than those applied for cell perfusion.

#### Influence of the magnetic field on cells in 2D culture

**Fig. 6:**
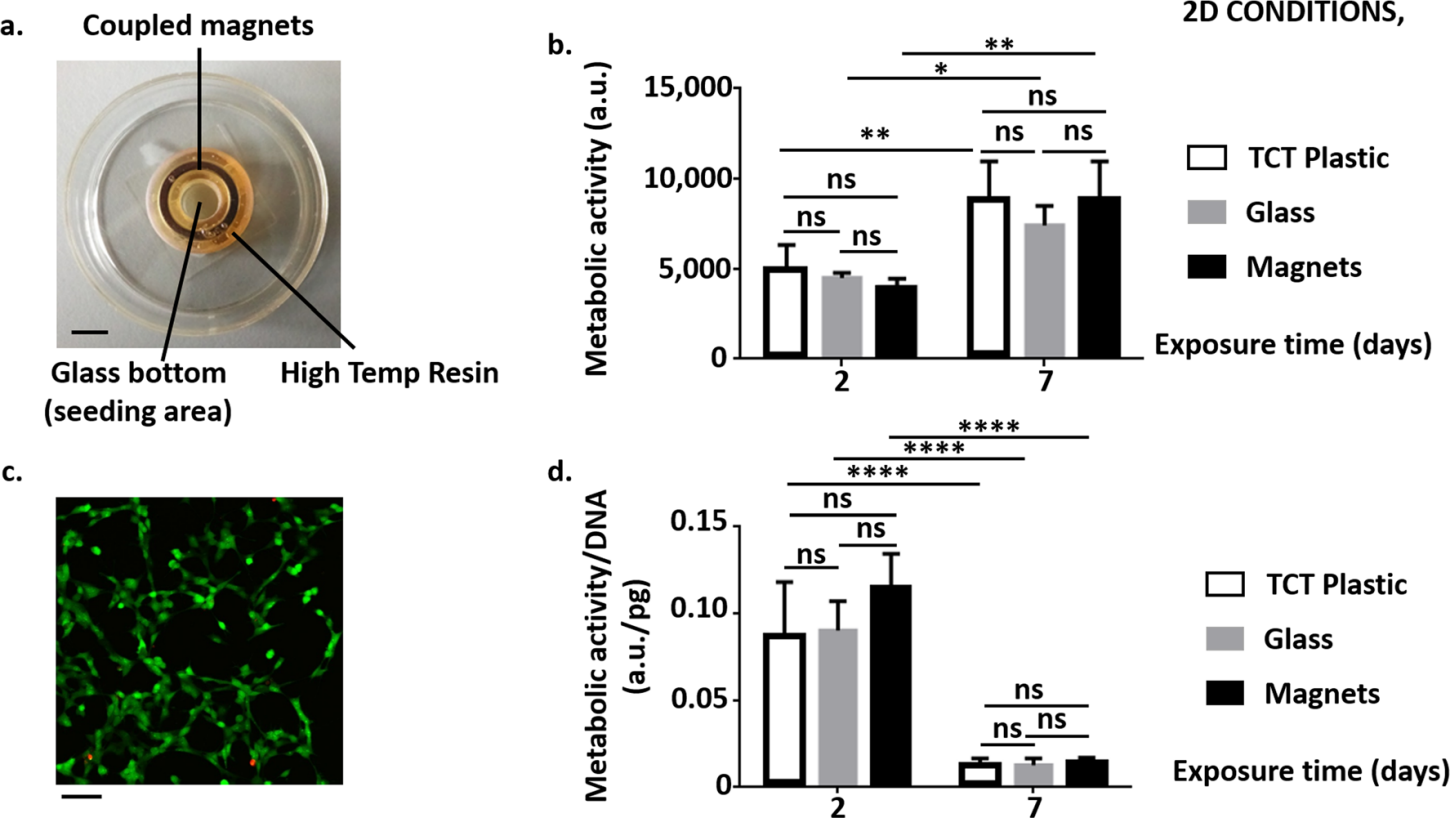
**Influence of the magnetic field on SH-SY5Y cells in 2D culture: specific metabolic activity. a** Experimental set-up to evaluate the effect of the magnetic field generated by the ring magnets of the MOAB on SH-SY5Y cells. Scale bar: 5 mm. **b** Metabolic activity of SH-SY5Y cells with time. For the short exposure time (2 days), we plated 93,750 cells/cm^2^; while for the long exposure time (7 days), we plated 31,250 cells/cm^2^. TCP indicates SH-SY5Y cells plated on standard tissue culture-treated dishes. **c** Example of a confocal microscopy image showing live (stained green by calcein AM) and dead (stained red by ethidium homodimer-1) SH-SY5Y cells after 48 h culturing in the absence of the magnetic field. Scale bar: 20 μm. **d** Specific metabolic activity (that is metabolic activity normalized with respect to the DNA content) of SH-SY5Y cells with time. For the shortest exposure time (2 days), we plated 93,750 cells/cm^2^; while for the other exposure times, we plated 31,250 cells/cm^2^. In all the graphs, we showed the results from resazurin assay (3 replicates/group). We reported the results as mean ± SD. We performed the statistical analysis with two-way ANOVA followed by Tukey’s multiple comparisons test. ns, *p* > 0.05; *, *p* < 0.05; **, *p* < 0.01; ****, *p* < 0.0001

Figure 6 shows the experimental set-up and the results related to the effect of the magnetic field on the viability, metabolic activity and gene expression of SH-SY5Y cells in 2D monolayer. We exposed the cells to the SMF one day after seeding because we noticed that its presence impaired cell adhesion, especially in the case of the lowest cell density (31,250 cells/cm^2^).

For both metabolic activity and specific metabolic activity, we did not find differences with controls on glass substrates (but also on standard tissue culture-treated dishes, TCP) cultured in the absence of the magnets (ns, *p* > 0.05), suggesting no effects of the magnetic field generated by the NdFeB magnets in the examined time window. Live/dead assay and confocal microscopy confirmed these results. Fig. 6c reports an example image: the great majority of SH-SY5Y cells cultured for 7 days in glass bottom dishes was alive (stained green by calcein AM). Only a few dead cells (stained red by ethidium homodimer-1) were visible.

**Fig. 7:**
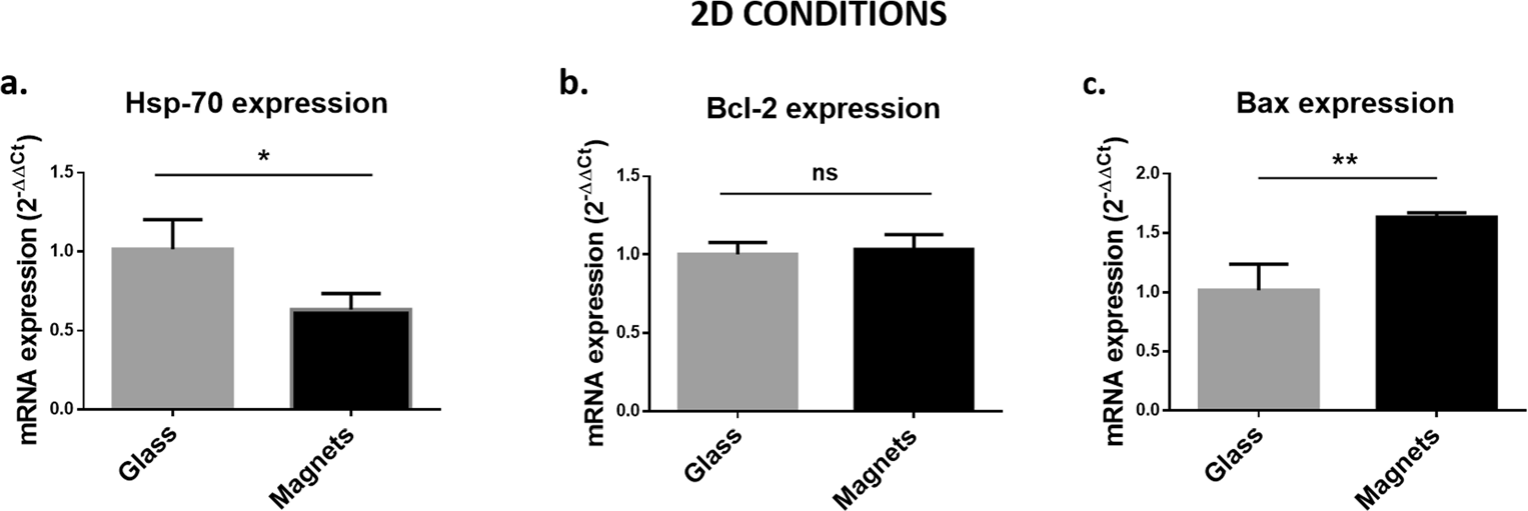
**Influence of the magnetic field on SH-SY5Y cells in 2D culture: mRNA expression levels. a** mRNA expression levels of heat shock protein-70 (Hsp70). **b** a) mRNA expression levels of Bcl-2. **c** mRNA expression levels of Bax. We reported the results as mean ± SD, 3 replicates/group. We performed the statistical analysis with t-test. ns, *p* > 0.05; *, *p* < 0.05; **, *p* < 0.01

Figure 7 reports the mRNA expression levels of the anti-apoptotic Hsp70 and Bcl-2 and the pro-apoptotic Bax after 7-days exposure. It shows a lower expression of Hsp70 (*, *p* < 0.05) and a higher expression of Bax (**, *p* < 0.01) in SH-SY5Y cells exposed to the magnetic field with respect to controls on glass substrates. We did not detect significant differences in the mRNA expression levels of Bcl-2 (ns, *p* > 0.05).

#### Influence of the magnetic field on cells in 3D culture

**Fig. 8:**
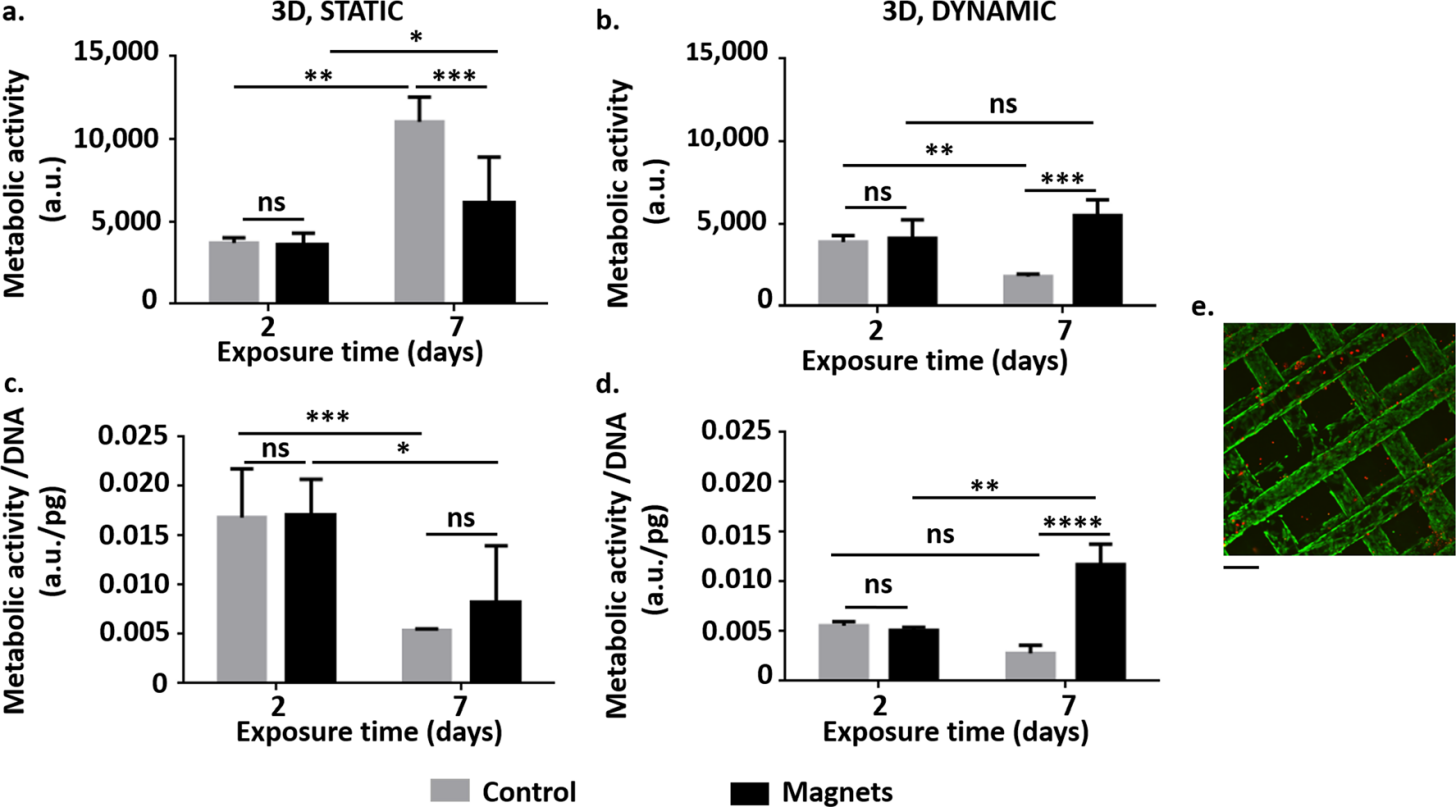
**Influence of the magnetic field on SH-SY5Y cells in 3D culture: specific metabolic activity. a** Metabolic activity of SH-SY5Y cells with time (in static conditions). **b** Metabolic activity of SH-SY5Y cells with time (in dynamic conditions). **c** Specific metabolic activity (that is metabolic activity normalized with respect to the DNA content) of SH-SY5Y cells with time (in static conditions). **d** Specific metabolic activity of SH-SY5Y cells with time (in dynamic conditions). In all the graphs, we showed the results from resazurin assay (3 replicates/group). We reported the results as mean ± SD. We performed the statistical analysis with two-way ANOVA followed by Tukey’s multiple comparisons test. ns, *p* > 0.05; *, *p* < 0.05; **, *p* < 0.01; ***, *p* < 0.001; ****, *p* < 0.0001. **e** Example of confocal microscopy images showing live (stained green by calcein AM) and dead (stained red by ethidium homodimer-1) SH-SY5Y cells after 48 h culturing in static conditions in the absence of the magnetic field. Results from a z-stack acquisition. Scale bar 20 μm

Figure 8 shows the results related to the effect of the magnetic field on the metabolic activity and specific metabolic activity of SH-SY5Y when in 3D scaffolds both in dynamic or static conditions.

After 2 days in static conditions, we did not observe effects on metabolic activity and specific metabolic activity. After 7 days, we measured a significant difference in metabolic activity (***, *p* < 0.001), but it was recovered when calculating the specific metabolic activity (ns, *p* > 0.05).

In dynamic conditions, again we observed a statistical difference (***, *p* < 0.001) in both metabolic activity and specific metabolic activity after 7 days of exposure to the SMF. However, the situation was reversed with respect to the static condition, with the cells exposed to magnetic field exhibiting greater values than controls.

Live/dead assay and confocal microscopy supported these results. Fig. 8e shows an example image: the great majority of SH-SY5Y cells cultured for 7 days in static conditions was alive (stained green by calcein AM). Only a few dead cells (stained red by ethidium homodimer-1) may be detected. Finally, confocal microscopy suggested a good degree of scaffold colonization, reporting the presence of living cells at least on three of the four layers.

**Fig. 9:**
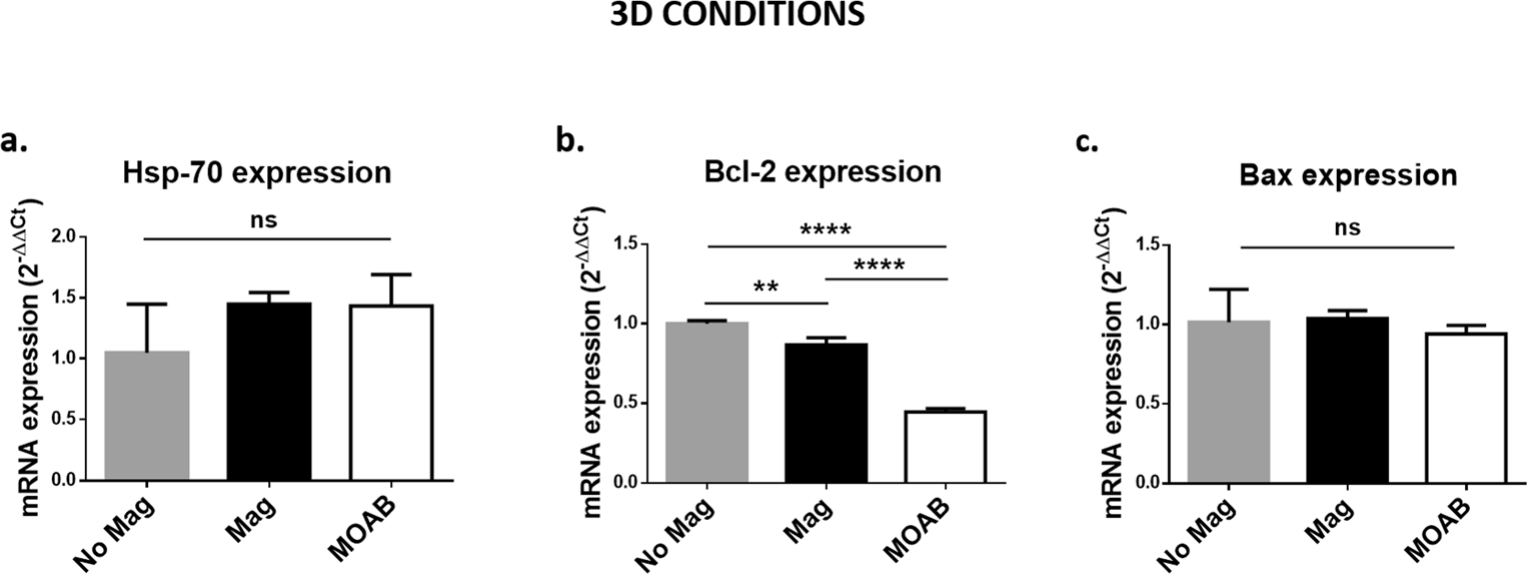
**Influence of the magnetic field on SH-SY5Y cells in 3D culture: mRNA expression levels. a** mRNA expression levels of heat shock protein-70 (Hsp70). **b** a) mRNA expression levels of Bcl-2. **c** mRNA expression levels of Bax. We reported the results as mean ± SD, 3 replicates/group. We performed the statistical analysis with one-way ANOVA followed by Dunnett’s multiple comparisons test. ns, *p* > 0.05; ***, *p* < 0.001; ****, *p* < 0.0001

Gene expression analyses (Fig. 9) showed different Hsp-70, Bcl-2 and Bax profiles between magnetic (Mag-static conditions; MOAB-dynamic conditions) and control samples (No Mag, static conditions) with respect to SH-SY5Y cells in 2D conditions. The expression levels of the anti-apoptotic Hsp70 did not decrease (ns, *p* > 0.05) and the expression levels of the pro-apoptotic Bax did not increase (ns, *p* > 0.05) in magnetic samples with respect to controls. However, the expression levels of Bcl-2 were significantly lower in the presence of the SMF (**, *p* < 0.01 for Mag samples; ****, *p* < 0.0001 for Mag samples), suggesting a stressful effect that does not reduce cell-specific metabolic activity.

## Discussion

This study aimed at assessing the effects of the SMF generated by the magnetic closure of the optimized prototype of a recently developed miniaturized bioreactor (Laganà and Raimondi 2012) on the metabolic activity, viability and gene expression of target genes (Hsp-70, Bcl-2 and Bax) in human SH-SY5Y neuroblastoma cells. Our results indicated that: 1) in the MOAB with magnetic closing, cells are exposed to non-zero values of SMF; 2) the maximum value of the SMF estimated with the 3D complete model (620 mT) is independent on the chamber position (lateral or central); 3) the maximum value of the SMF estimated by the axial-symmetric model (570 mT) is reduced by less than 10% with respect to the SMF estimated by the 3D complete model (620 mT); 4) the SMF generated by the NdFeB ring magnets does not reduce cell-specific metabolic activity after 48 h and 7 days in 2D and 3D conditions (both static and dynamic); 5) the SMF exerts a stressful effect in 2D conditions, but it decreases in 3D conditions (both static and dynamic).

Due to the very limited experimental accessibility of our miniaturized system to measurement of the magnetic field, we used a numerical analysis to predict the magnetic field generated by the magnetic rings. In fact, the culture chambers are very small (9 mm^3^) and it was not possible to find a suitable magnetometer able to avoid any influence on the read-outs while measuring. Moreover, a magnetometer is not able to show the distribution of the magnetic field. Peng and co-workers described an analytical method to calculate the magnetic field only for the central axis of an axially magnetized ring (Peng et al. 2004), but to our knowledge no analytical methods calculating the distribution of the magnetic field around a magnetic ring exist. Instead of NdFeB, we considered neodymium as the material for the domains of magnetic coupling because it is the strongest magnetic material of the alloy. In fact, neodymium magnets have a coercivity of about four orders of magnitude greater than that of a material with only a ferrous component.

Taking as a reference the intensity of Earth magnetic field (25–65 μT), we estimated greater values (320–620 mT) in the geometrical center of the rings. Therefore, the assessment of a possible influence of the magnetic field on cell constructs was necessary.

In the last few years, several studies have focused on the influence of SMFs at the cellular level. They have reported a dependence on magnetic field intensity, exposure time, cell type and density, but results are contradictory and a comparison is difficult because of the differences in experimental parameters and read-outs (e.g. cell number, cell metabolic activity, orientation of intracellular components, DNA transcription). Since changes in cell cycle or at intracellular level (e.g. growth factor signaling, DNA transcription) have a direct impact on cell viability and functions (Albuquerque et al. 2016), in this work we focused on cell metabolic activity, number and gene expression. For 2D studies, we preferred dishes with glass bottom to standard polystyrene microplates because the latter does not allow for cell observation by confocal microscopy. Since the multi-layer nickel-copper-nickel plating may not be sufficient to prevent the corrosion of neodymium magnets for all applications, we covered NdFeB magnets with a protective layer of High Temp Resin after assessing its biocompatibility with SH-SY5Y cells (Online Resource 1).

In both 2D and 3D conditions, we observed that the SMF does not reduce cell-specific metabolic activity after 48 h and 7 days of exposure. For cells in 2D conditions, Miyakoshi (2005) published that most of studies reported no significant effects of SMF on cell viability. Similarly, Romeo and co-workers (Romeo et al. 2016) reported lack of effects on cell viability when exposing MRC-5 human lung fibroblasts to a SMF of 370 mT. Oppositely, biological effects were observed when the magnetic field was coupled to stimuli like x-irradiation and in the literature there is evidence that SMFs can affect several endpoints when in the mT range. For SH-SY5Y cells exposed for 24 h to 2.2 mT SMF, Calabrò and colleagues (Calabrò et al. 2013) observed a decrease of membrane mitochondrial potential up to 30% and an increase in the production of reactive oxygen species. On the other end, Stolfa and co-workers (Stolfa et al. 2007) reported an increase in viability for human chondrocytes exposed for 72 h to a SMF of 600 mT. To our knowledge, this is the first study examining the effects of the SMF on SH-SY5Y cells in 3D conditions, therefore no direct comparison with data in the literature is possible.

We deepened our investigations with genetic assays and analysed the expression profile of Hsp70, Bcl-2 and Bax. Hsp70 can be activated when cells are exposed to particular stressors and it represents one of the actors of the apoptosis regulation. Bcl-2 is an anti-apoptotic protein that inhibits cell death by regulating the Bcl-2-regulated apoptotic pathway, while Bax has a pro-apoptotic role in the events triggering programmed cell death. We selected these genes from a previous work (Tenuzzo et al. 2009). Here, the authors evaluated the biological effects of a SMF of 6 mT on aged human lymphocytes. Similarly to Tenuzzo and colleagues, we demonstrated that the expression of Hsp70 decreases and the expression of Bax increases after 7-day exposure to our SMF in 2D conditions. Together with the results from cell metabolic activity, these results suggest that the SMF generated by the NdFeB ring magnets is not stressful enough to reduce cell-specific metabolic activity. In 3D dynamic conditions (MOAB samples), Hsp70 and Bax expression was comparable with controls (No Mag samples), suggesting a reduction of this stressful effect. The significant lower levels of Bcl-2 in SH-SY5Y cells subjected to the SMF suggested a possible weak stressed condition that should be further investigated.

The hydraulic characterization performed in the present study indicated that both the magnetically and snap-fit lockable MOABs may stand pressure values higher than those usually involved in cell perfusion experiments, where maximum flow rates are in the order of μL/min. However, at higher pressures the flow rate offered by the bioreactor with magnetic closing is significantly greater. This is probably due to the better hydraulic sealing of the magnetic coupling with respect to the simple snap-fit closure for mechanical interference. Cell proliferation inside the scaffolds increases the mechanical resistance to the fluid flow within the chambers. To avoid leakage and guarantee a suitable perfusion to cell constructs during experiments up to 7 days, we inserted screws and cable ties on non-magnetically bioreactors. Despite these precautions, the specific metabolic activity was significantly lower than in magnetic dynamic samples (*p* < 0.0001), suggesting that our snap-fit bioreactors are not suitable for perfusion experiments longer than 48 h. For these reasons, we did not perform genetic assays on these samples.

However, the MOAB with snap-fit closure would remain a forced choice for magnetic particle tracking, apart from their dimensions and charge.

## Conclusions

We predicted the values and distribution of the SMF generated by the NdFeB ring magnets in the culture chambers of the MOAB by a numerical analysis, indicating that the intensity of the magnetic field has a quadratic decay trend along the radial direction of the culture chamber, with very low values affecting all the central areas of the cultured scaffold. Furthermore, the maximum intensity of the magnetic field generated in each chamber of the bioreactor is not influenced by the adjacent chamber(s). The hydraulic characterization recorded a significantly higher leakage for non-magnetically lockable MOABs compared to the standard magnetic ones. We found that after 2 and 7 days of cell exposure to the SMF generated by the magnetic closure of the MOAB, the cell-specific metabolic activity of SH-SY5Y cells is not reduced, neither in 2D monolayer, nor in 3D static culture, nor in the MOAB. Instead, the expression levels of Hsp70, Bcl-2 and Bax measured in cells at seven culture days shows that this level of SMF exerts a stressful effect in 2D monolayer, which decreases to a negligible level in 3D static culture, and in the MOAB. Taken together, these results suggest that MOAB device with magnetic closure might be potentially used also as the basic functional unit of the ERC “MINERVA” multi-organ bioengineered platform.

## Electronic supplementary material

ESM 1 (DOC 980 kb)
