# Supplementary material for: Influence of the static magnetic field on cell response in a miniaturized optically accessible bioreactor for 3D cell culture
Source: Biomed Microdevices. 2019 Mar 13;21(1):29. doi: 10.1007/s10544-019-0387-8 (PMC6451746; doi:10.1007/s10544-019-0387-8)
Supplement: Supplementary file 1 — (DOC 980 kb) [file 10544_2019_387_MOESM1_ESM.doc]

**Article title:** Influence of the static magnetic field on cell response in a miniaturized optically accessible bioreactor for 3D cell culture

**Journal name:** Biomedical Microdevices

**Author names:**

Luca Izzo^*,#^, Marta Tunesi^#^, Lucia Boeri, Matteo Laganà, Carmen Giordano, Manuela Teresa Raimondi

# These authors contributed equally to this work

***Correspondence to:**

L. Izzo

Department of Chemistry, Materials and Chemical Engineering “G.Natta”

Politecnico di Milano, P.za Leonardo da Vinci 32

20133, Milan, Italy

E-mail: [luca.izzo@polimi.it](mailto:luca.izzo@polimi.it)

Fax: +39.02.2399.4776

**Electronic Supplementary Material 1:** Assessment of the biocompatibility of High Temp Resin with SH-SY5Y cells

**Materials and Methods**:

To assess the biocompatibility of High Temp Resin, we cut samples with a hole saw (diameter: 5 mm; (6.52·10^-2^ ± 7.63·10^-3^) g), placed in 48-well microplates (Corning, Corning, NY, USA) and dipped with 375 µL culture medium. After 24h- and 7 day-incubation, we collected the supernatants and stored at 4°C.

We plated SH-SY5Y cells (93,750 cells/cm^2^) in 96-well microplates. The following day we replaced culture medium with 100 µL supernatant, eventually diluted with fresh medium (D1 = 1:100 v/v; D2 = 1:50 v/v; D3 = 1/10 v/v; D4 = not diluted). After 24h incubation, we evaluated cell metabolic activity by resazurin assay. We normalized the results with respect to controls in standard medium and reported as mean ± SD.

**Results**:

Following incubation with the supernatants collected after 24h, bright-field images (Online Resource 1, fig. a), showed that at all the dilutions tested SH-SY5Y cells extended long neuritis and their morphology was similar to CTRL. This result was supported by resazurin assay (Online Resource 1, fig. b), reporting that the metabolic activity of SH-SY5Y cells was comparable to CTRL for all the conditions (ns, *p*>0.05). Following incubation with the supernatants collected after 7 days (Online Resource 1, fig. c), the metabolic activity of SH-SY5Y cells was comparable to CTRL only for the dilutions D1, D2 and D3 (ns, *p*>0.05). When we cultured SH-SY5Y cells in the supernatants not diluted with fresh medium (D4), their metabolic activity was significantly lower than CTRL (****, *p*<0.0001). However, our custom-made setup does not allow for the occurring of this last condition. In fact, we cultured SH-SY5Y cells in the presence of a greater volume of culture medium (about 2 mL), that was refreshed every 2 days.

| 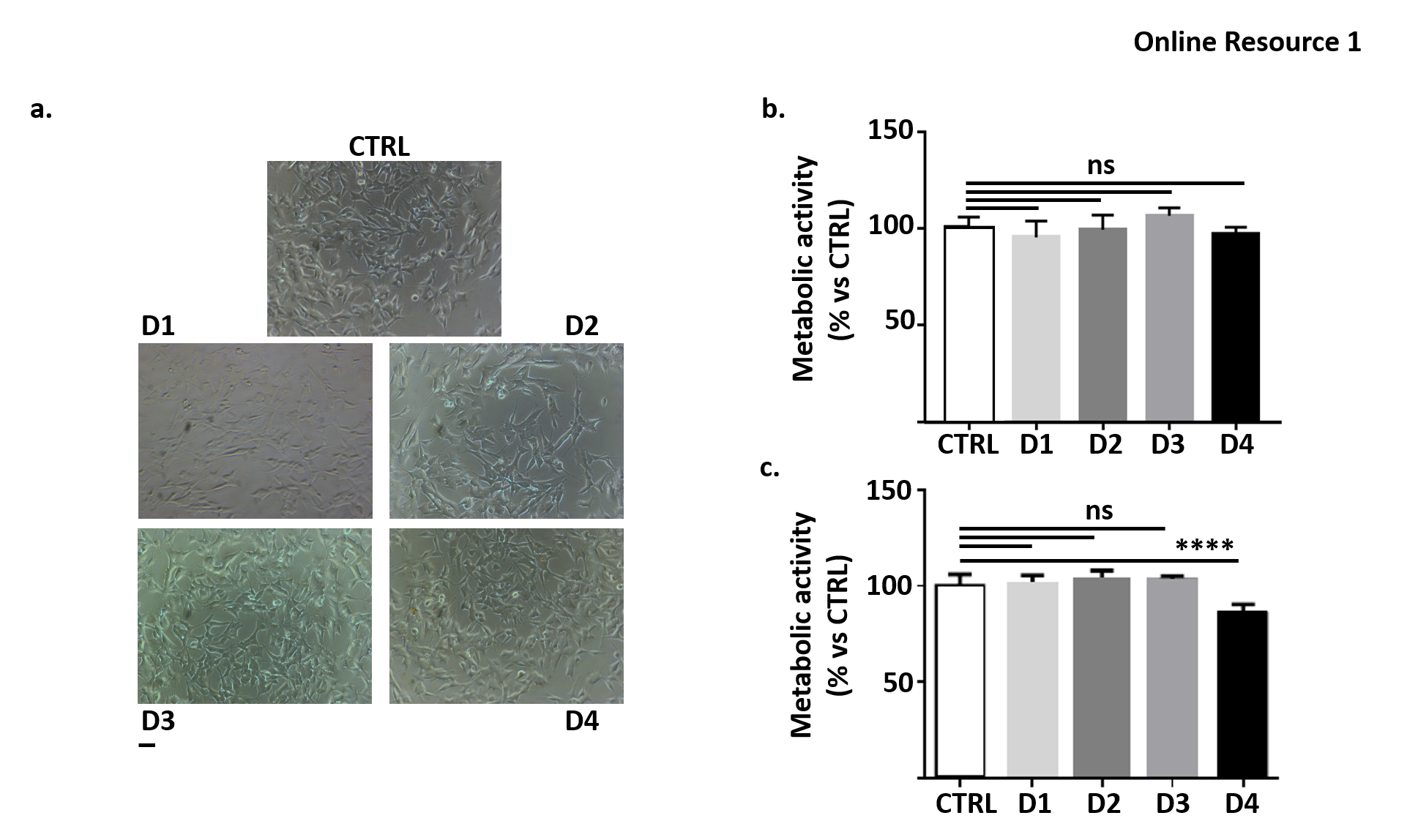 |
| --- |
| **Online Resource 1**: a) Morphology of SH-SY5Y cells after exposure to supernatants collected after 24h incubation from High Temp Resin samples. Scale bar: 10 µm. Metabolic activity of SH-SY5Y cells after 24h exposure to supernatants collected after 24h-incubation (b) or 7 day-incubation (c) from High Temp Resin samples. We diluted supernatants with medium: D1 = 1:100 v/v; D2 = 1:50 v/v; D3 = 1/10 v/v; D4 = not diluted. Results from resazurin assay, 7 replicates/group. Results are mean ± SD. We performed the statistical analysis with respect to controls in standard medium (CTRL) with one-way ANOVA followed by Dunnett's multiple comparisons test. ns, *p*>0.05; ****, *p*<0.0001. |
